# Proceedings of the American Pharmaceutical Association, at Its 12th Annual Meeting, Held in Cincinnati, Sept. 1864

**Published:** 1865-03

**Authors:** 


### Book Notices.

PROCEEDINGS OF THE AMERICAN PHARMACEUTICAL ASSOCIATION, at its Twelfth Annual Meeting, held in Cincinnati, O., September, 1864. Also, the REVISED CONSTITUTION AND ROLL OF MEMBERS. Philadelphia: MERRIHEW & SON, Printers, 243 Arch Street. 1864.

This is a neatly bound volume, of 355 pages. In addition to a full record of the proceedings of the last Annual Meeting of the American Pharmaceutical Association, it contains reports and papers on the following subjects, viz.:—Report on the Progress of Pharmacy; on the Drug Market; on the Preparations of Cinchona; on the Extraction and Preservation of Lard; on the Solubility of Camphor in Water; on the Aqueous Extract of Jalap; on the Pharmaceutist as a Merchant; on the Sweet-Gum Tree; on Tobacco; Preparation and Dispensing of Plasters; Bitter Wine of Iron; Glycerin—its Mission; on Spatulas, Stirring Rods, &c.; on a Test for the Resin of Cannabis Indica; on Pyrethrum Roseum; The Press; on the Odor of Commercial Tannic Acid; on the Preservation of Garlic; on a Brandy Substitute; on Capsicum; Remarks on Dialysis; on Southern Prickly-Ash Bark; a Systematic Course of Study and Manipulation for Students of Pharmacy; on the Assay of French Brandy and Whiskey; on the Quality of Sherry Wine of our Commerce, and its Assay; on Some Medicinal Spirits; Practical Observations on the Manufacture of Æthereum Oleum; Plan and Description of Balance for Apothecaries' Uses; Description of a New Balance for Use without Weights, &c.

Many of the foregoing articles are short, but they are all of more or less interest and importance, and they constitute a volume which should be in the hands of every Druggist and Apothecary throughout the country.

While it is of special interest to the Druggist, it is also well worthy of perusal by the Practising Physician. Copies of the work may be obtained of E. H. SARGENT, corner of Randolph and State Streets, Chicago. Price $1,50.

